# Machine Learning Method for Fatigue Strength Prediction of Nickel-Based Superalloy with Various Influencing Factors

**DOI:** 10.3390/ma16010046

**Published:** 2022-12-21

**Authors:** Yiyun Guo, Shao-Shi Rui, Wei Xu, Chengqi Sun

**Affiliations:** 1 State Key Laboratory of Nonlinear Mechanics, Institute of Mechanics, Chinese Academy of Sciences, Beijing 100190, China; 2 School of Engineering Science, University of Chinese Academy of Sciences, Beijing 100049, China; 3 Beijing Key Laboratory of Aeronautical Materials Testing and Evaluation, Beijing Institute of Aeronautical Materials, Beijing 100095, China

**Keywords:** machine learning, nickel-based superalloy, fatigue strength prediction, temperature, stress ratio

## Abstract

The accurate prediction of fatigue performance is of great engineering significance for the safe and reliable service of components. However, due to the complexity of influencing factors on fatigue behavior and the incomplete understanding of the fatigue failure mechanism, it is difficult to correlate well the influence of various factors on fatigue performance. Machine learning could be used to deal with the association or influence of complex factors due to its good nonlinear approximation and multi-variable learning ability. In this paper, the gradient boosting regression tree model, the long short-term memory model and the polynomial regression model with ridge regularization in machine learning are used to predict the fatigue strength of a nickel-based superalloy GH4169 under different temperatures, stress ratios and fatigue life in the literature. By dividing different training and testing sets, the influence of the composition of data in the training set on the predictive ability of the machine learning method is investigated. The results indicate that the machine learning method shows great potential in the fatigue strength prediction through learning and training limited data, which could provide a new means for the prediction of fatigue performance incorporating complex influencing factors. However, the predicted results are closely related to the data in the training set. More abundant data in the training set is necessary to achieve a better predictive capability of the machine learning model. For example, it is hard to give good predictions for the anomalous data if the anomalous data are absent in the training set.

## 1. Introduction

Fatigue damage is one of the main factors for the failure of mechanical parts. Therefore, the accurate design of the fatigue strength or fatigue life is an urgent problem in the industrial field. However, there are many factors affecting fatigue performance, such as stress ratios, loading frequencies, temperatures, surface roughness, residual stresses, defects of materials, etc. [1,2,3,4,5,6,7]. For example, fatigue life usually increases with the increase in the stress ratio at the same maximum stress [8,9,10,11,12,13]. The nickel-based superalloy does not present a monotonous trend with the increase in temperature, which reaches peak value at a certain temperature [14,15,16]. The defect tends to reduce the fatigue performance of metallic materials, and the fatigue strength often decreases with the increase in the defect size [17,18,19].

Machine learning is a method that builds models based on the data and uses models to predict and analyze the data. The deep learning developed in recent years has been widely used in various fields due to its strong learning and perceptual capabilities, such as games [20,21,22], drug design [23,24,25] and human health testing [26,27,28], which has played an important role in dealing with the association or influence of complex factors. Some results have also been available on the application of machine learning in the field of fatigue [29,30,31,32,33,34]. For example, Iacoviello et al. [29] studied the effect of the stress ratio *R* on the fatigue crack growth resistance of PM duplex stainless steel through a model based on the artificial neural network (ANN), and predicted the fatigue crack propagation with a high degree of coincidence. Durodola et al. [30] used the ANN method to evaluate the effect of mean stress on the fatigue life of metal alloys. Their results showed that the ANN method had higher resolution and consistency compared with the existing methods. Yan et al. [31] proposed a hybrid model to predict the fatigue strength of steels based on a modified bagging method, which showed great potential in improving the prediction accuracy of the fatigue strength.

The factors influencing fatigue behavior are complicated, and the associated mechanism is not completely clear. Therefore, it is difficult to correlate well the influence of various factors on fatigue life or fatigue strength. Taking the effects of temperature and stress ratio on fatigue strength as an example, it is hard to establish a traditional math model to reflect well the coupling effect of temperature and stress ratio on fatigue strength at different fatigue lifetimes. Machine learning, meanwhile, is only the association of numbers in an algorithm, which does not need to establish explicit expressions. This provides a way to describe the influence of multiple factors.

The Ni-based superalloy GH4169 is one of the main materials for the manufacture of advanced engine turbine disks and turbine blades, which is subjected to fatigue loadings in service. In this study, three typical machine learning methods, the gradient boosting regression tree (GBRT) model, the long short-term memory (LSTM) model and the polynomial regression model with ridge regularization (PRRR), are used to investigate the fatigue strength prediction of the Ni-based superalloy GH4169 incorporating multiple influencing factors. The GBRT model and the PRRR belong to the category of machine learning, while the LSTM model belongs to the deep neural network architecture. GBRT is an integrated model, while LSTM and PRRR are single models. The aim of this paper is to examine the predictive ability of the machine learning method in dealing with the influence of various factors on fatigue performance. By dividing different training sets and testing sets, it is found that the predicted results by the machine learning method are closely related to the training set. In the absence of the anomalous data, all three models give reasonable predictions. If the anomalous data are not in the training set, it is hard to predict the anomalous data. The predictive capability is also compared for the three machine learning methods. It shows that the PRRR gives the best predicted results of fatigue strength.

## 2. Experimental Data and Pre-Processing

### 2.1. Experimental Data

**Figure 1:**
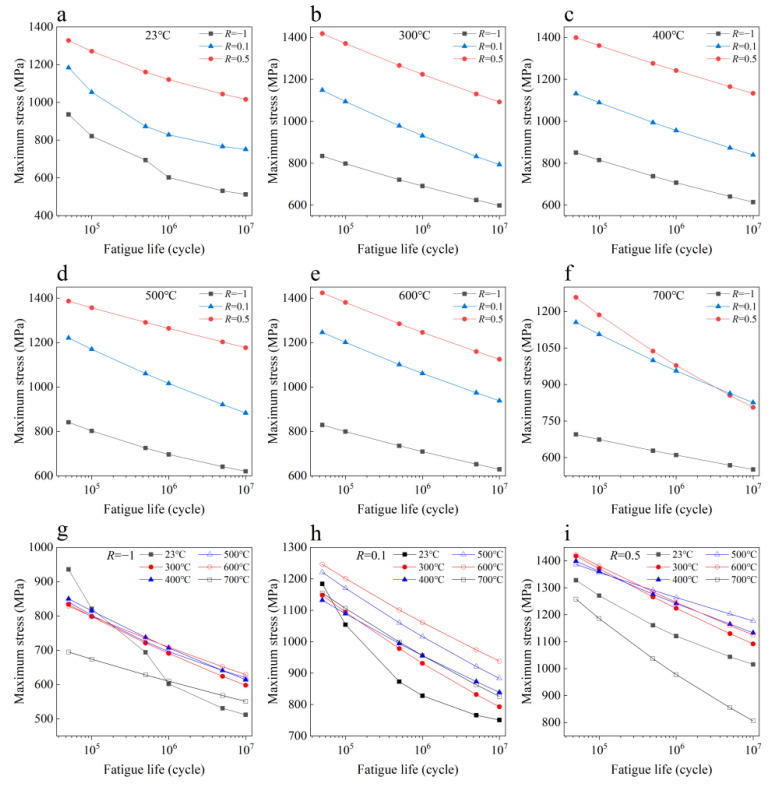
S-N data of nickel-based superalloy at different temperatures and stress ratios: (**a**–**f**) Different stress ratios at 23 °C, 300 °C, 400 °C, 500 °C, 600 °C, 700 °C, respectively; (**g**–**i**) Different temperatures at *R* = −1, *R* = 0.1 and *R* = 0.5, respectively.

The data used in this study are the fatigue strengths of a nickel-based superalloy GH4169 under different temperatures, stress ratios and fatigue lifetimes from Ref. [35]. The total experimental data are 108, and it is composed of 18 data at each temperature. Figure 1 shows the relationship between the fatigue strength and the fatigue life of this material at different temperatures and stress ratios. It is seen from Figure 1a–f that the fatigue strength in terms of the maximum stress at a given fatigue life increases with the increase in the stress ratio at the same temperature except for the fatigue strength of 5 × 10^6^ cyc and 10^7^ cyc at 700 °C. Figure 1g–i indicates that the effect of temperature on fatigue strength is related to the stress ratio and fatigue life. At the stress ratio *R* = −1, the difference of fatigue strength is small when the temperature ranges from 300 °C to 600 °C at the same fatigue life. The fatigue strength at 700 °C is obviously lower than the fatigue strength at 300 °C to 600 °C at the same fatigue life. With the increase in fatigue life, the fatigue strength at room temperature (23 °C) descends sharply compared with the fatigue strength at high temperatures. The fatigue strength at room temperature is lower than the one at high temperature at the fatigue life of 10^6^ cyc, 5 × 10^6^ cyc and 10^7^ cyc. At the stress ratio *R* = 0.1, the fatigue strength generally increases with an increase in temperature at the same fatigue life when the temperature ranges from 23 °C to 600 °C, while the fatigue strength at 700 °C is almost the same as the fatigue strength at 400 °C. At the stress ratio *R* = 0.5, the difference of the fatigue strength is often not large when the temperature ranges from 300 °C to 600 °C, while the fatigue strength at 700 °C is obviously lower than the fatigue strength at 300 °C to 600 °C. The fatigue strength at room temperature is lower than the fatigue strength at 300 °C to 600 °C but higher than the one at 700 °C.

### 2.2. Data Pre-Processing

The predictive ability of the PRRR and LSTM model could be affected by the inconsistent dimensions between different features and the large numerical difference in the data set. Therefore, in order to improve the accuracy of the models, the MaxMinscaler normalization is performed on the data according to Equation (1) before constructing the PRRR and LSTM model, i.e., the input characteristics (temperature, stress ratio, fatigue life) is mapped to the interval [–1, 1], and the output fatigue strength is mapped to the interval [0, 1].

(1) $$x^{\prime }=a+\frac{ [x-\min\left(x\right)]\left(b-a\right)}{\max\left(x\right)-\min\left(x\right)}$$

where *x* and *x′* are the values before and after feature scaling, respectively, Max(*x*) and Min(*x*) are the maximum and minimum values of features, respectively, *a* and *b* are real numbers that adjust the range of the zoom interval [*a*, *b*].

## 3. Machine Learning Models

### 3.1. Gradient Boosting Regression Tree (GBRT) Model

The GBRT [36] is an ensemble algorithm that combines several weak learners into one strong learner. It adds predictors to the ensemble step by step, each of which corrects its preamble, so that the new predictor fits the residual of the previous predictor. The process of this algorithm is as follows:

(1) Initialization:

(2) $$f_{0}\left(x\right)=\arg\underset{c}{\min}\overset{N}{\underset{i}{\sum }}L\left(y_{i},c\right)$$

(2) For *m* = 1, 2, …, *M*:

(a) For *i* = 1, 2, …, *N*, calculate:

(3) $$r_{mi}=-{ [\frac{\partial L\left(y_{i},\;\;f\left(x_{i}\right)\right)}{\partial f\left(x_{i}\right)}]}_{f\left(x\right)=f_{m-1}\left(x\right)}$$

(b) Fit a regression tree to *r_mi_* to get the leaf node region *R_mj_* of the *m*-th tree, *j* = 1, 2, …, *J*.

(c) For *j* = 1, 2, …, *J*, calculate:

(4) $$c_{mj}=\arg\underset{c}{\min}\underset{x_{i}\in R_{mj}}{\sum }L\left(y_{i},f_{m-1}\left(x_{i}\right)+c\right)$$

(d) Update the weak learner *f_m_*(*x*):

(5) $$f_{m}\left(x\right)=f_{m-1}\left(x\right)+\overset{J}{\underset{j=1}{\sum }}c_{mj}I\left(x\in R_{mj}\right)$$

(3) Get the regression tree:

(6) $$\overset{\wedge }{f}\left(x\right)=f_{M}\left(x\right)=\overset{M}{\underset{m=1}{\sum }}\overset{J}{\underset{j=1}{\sum }}c_{mj}I\left(x\in R_{mj}\right)$$

Firstly, the GBRT algorithm is initialized at the first step to estimate the constant value that minimizes the loss function *L*. Then, the negative gradient *r_mi_* of the loss function is calculated as the approximate value of the residual in step 2(a). In step 2(b), the leaf node area of the regression tree is estimated to fit the approximate value of the residual error. In step 2(c), the value of the leaf node area is estimated by the linear search to minimize the loss function. The regression tree is updated continuously in step 2(d). Finally, the final model is output in step 3.

The GBRT model is usually sensitive to the setting of parameters. The appropriate setting of parameters can improve the generalization performance of the model. In this study, the parameters “max_depth” and “learning _rate” are 4 and 0.1, respectively, so as to avoid the over-complexity of the model and improve its learning ability. In addition, the optimal number of trees is obtained by adopting the early stop method, i.e., training a GBRT model with *n* trees at first, and then measuring the verification error of each stage to find the optimal number of trees, and finally using the optimal number of trees to retrain the model.

### 3.2. Long Short-Term Memory (LSTM) Model

**Figure 2:**
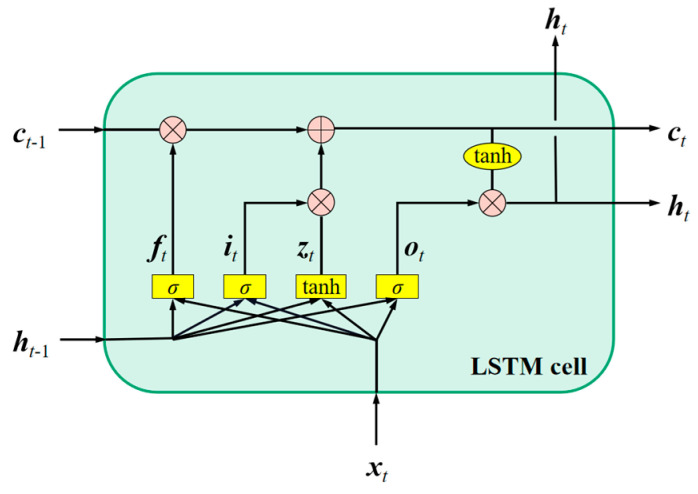
Basic architecture of LSTM unit. *h_t_* and *c_t_* denote short-term state and long-term state, respectively. *f_t_*, *i_t_* and *o_t_* represent forgetting gate, inputting gate and outputting gate, respectively, which controls the path of information transmission. *z_t_* is the candidate state obtained by the nonlinear function, *σ* is Logistic function, tanh is hyperbolic tangent function. ⊗ and ⊕ denote the multiplication and addition of the corresponding elements, respectively. The detailed information for the LSTM unit can be referred to Ref. [44].

The LSTM [37] model allows information to selectively affect the state of each moment in the network by introducing three “gate” structures: inputting gate, forgetting gate and outputting gate. The basic architecture of the LSTM unit is shown in Figure 2. The long-term state ***c****_t−_*_1_ first passes through a forgetting gate, loses some memories, and then adds some new memories selected by the inputting gate through an additional operation. Results of ***c****_t_* are directly output without any further conversion. After the additional operation, the long-term state is copied and transmitted through the hyperbolic tangent function. Then, the result is filtered by the outputting gate, and the short-term state ***h****_t_* is generated. The LSTM model can learn to identify important inputs, store them in a long-term state, keep or extract it when it is needed. The LSTM network has been widely used in machine translation, speech recognition and some other fields with its excellent data processing ability, and has achieved amazing success [38,39,40,41,42,43].

In the construction of the long short-term memory network, the number of neurons in the LSTM layer decreases layer by layer to form a pyramid. The reason for this construction is that many low-level features can be merged into fewer high-level features, and the attention is shifted to the relationships that have a significant relevance to the output. Moreover, considering that the output fatigue strength is positive, the Relu activation function is adopted in the output layer to ensure that the network can model the complex nonlinear phenomenon and learn efficiently.

### 3.3. Polynomial Regression Model with Ridge Regularization (PRRR)

The polynomial regression with ridge regularization is an introduction of the ridge regularization on the basis of polynomial regression. Ridge regression is a regularized version of the linear regression whose cost function can be expressed by Equation (7). By adding a regularization term, $\alpha \overset{n}{\underset{1}{\sum }}\theta_{i}^{2}$, not only is the algorithm forced to fit the data, but also the weight of the model is constrained to achieve the purpose of regularization

(7) $$J\left(\theta \right)=MSE\left(\theta \right)+\alpha \frac{1}{2}\overset{n}{\underset{i=1}{\sum }}\theta_{i}^{2}$$

where *θ_i_* is the *i*-th model parameter (including the bias term *θ*_0_ and the feature weight *θ*_1_, *θ*_2_*,* …, *θ_n_*), *MSE*(*θ*) is the root mean square error and it is the cost function of the linear regression, *α* is the hyperparameter of the model and it controls the degree of regularization of the model. If *α* = 0, the ridge regression is only the linear regression. If α is very large, all weights are very close to 0 in the end. In this study, the parameters’ “degree” and *α* in PRRR are 10 and 0.05, respectively.

## 4. Results and Discussion

The experimental results in Figure 1 indicate that the fatigue strength at 700 °C is anomalous with the variation of the temperature compared with the other tested temperatures. Here, the fatigue strength data at 700 °C are seen as anomalous data. To examine the influence of data in the training set on the generalization performance of the machine learning model, four different cases of training sets and testing sets are considered for the fatigue strength data of the nickel-based superalloy, as shown in Table 1. In cases 1 and 2, the anomalous data are in the testing sets, and the predictive ability of the machine learning model for the anomalous data is examined. In cases 3 and 4, the anomalous data are absent in the testing sets, and the effect of anomalous data on the predicted results is investigated. In this study, all the machine learning models are implemented in Python-based programs. The temperature, stress ratio and fatigue life are input variables, and fatigue strength is the output variable.

**Figure 3:**
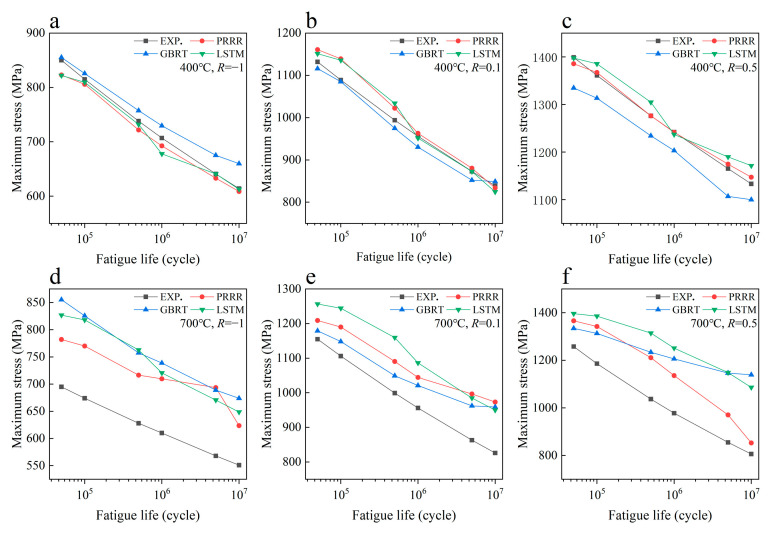
Predicted results of different machine learning models for the fatigue strength of the nickel-based superalloy under different conditions in case 1: (**a**) 400 °C, *R* = −1; (**b**) 400 °C, *R* = 0.1; (**c**) 400 °C, *R* = 0.5; (**d**) 700 °C, *R* = −1; (**e**) 700 °C, *R* = 0.1; (**f**) 700 °C, *R* = 0.5. (EXP. represents the experimental data).

**Figure 4:**
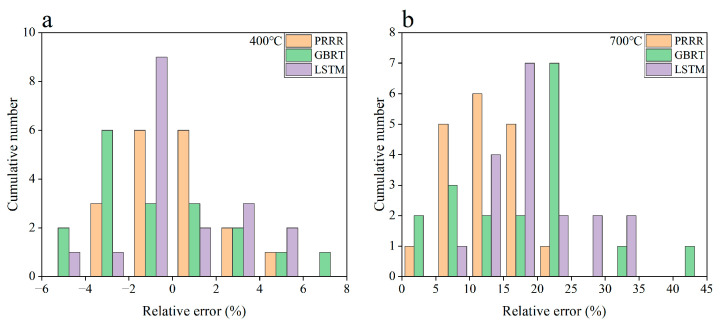
Relative errors of the predicted results to the experimental data for the different machine learning models in Figure 3: (**a**) 400 °C; (**b**) 700 °C. The range of relative errors is the same between the same numbers on the horizontal axis.

Figure 3 shows the predicted results of the above three machine learning models for the fatigue strength of the nickel-based superalloy at different temperatures and stress ratios in case 1. Figure 4 shows the relative errors of the predicted results to the experimental data for the different machine learning models. It is seen from Figure 3a–c that all the three machine learning models show good predictions for the fatigue strength under different stress ratios at 400 °C. The relative errors of the predicted results to the experimental data are all within 8% for the three machine learning models, as shown in Figure 4a, while for the fatigue strengths at 700 °C, it gives poor predictions for the three machine learning models (Figure 3d–f). The relative errors of a few predicted data are more than 30% for the GBRT and LSTM models, as shown in Figure 4b. The PRRR is better than the GBRT and LSTM models. The relative errors of the predicted results are all less than 25% for the PRRR.

**Figure 5:**
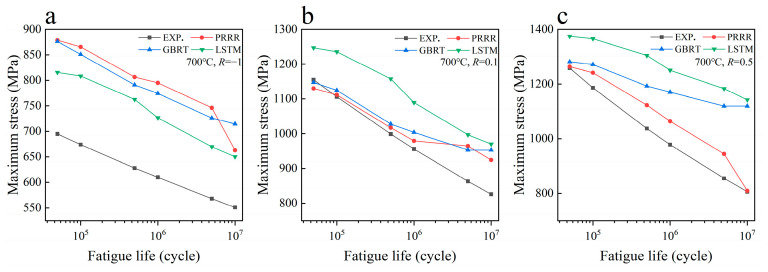
Predicted results of different machine learning models for the fatigue strength of the nickel-based superalloy in case 2: (**a**) 700 °C, *R* = −1; (**b**) 700 °C, *R* = 0.1; (**c**) 700 °C, *R* = 0.5.

**Figure 6:**
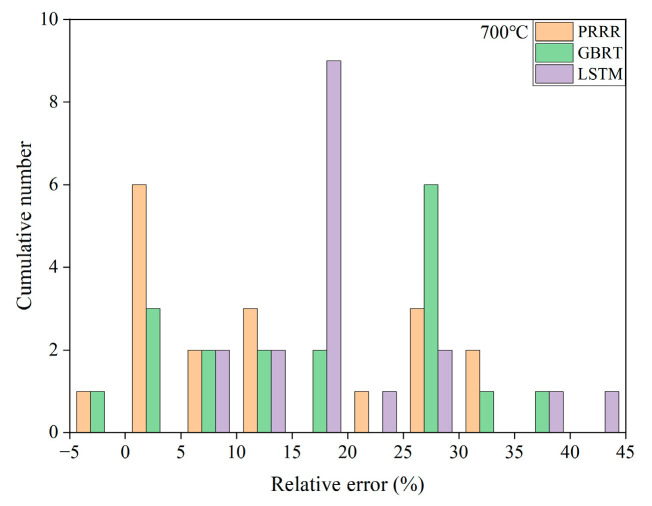
Relative errors of the predicted results to the experimental data for the different machine learning models in Figure 5. The range of relative errors is the same between the same numbers on the horizontal axis.

Figure 5 shows the predicted results of different machine learning models for the fatigue strength of the nickel-based superalloy in case 2. Compared with the results in Figure 3, the fatigue strength data at 400 °C are added in the training set for the predicted results in Figure 5. Figure 6 shows the relative errors of the predicted values to the experimental data for the different machine learning models in Figure 5. A comparison of the relative errors of the predicted results by the different training sets in Figure 4b with Figure 6 shows that the predictive ability is not improved for all three machine learning models by increasing the normal data in the training set. There is still a large deviation between the predicted values and the experimental ones. The results in Figure 3, Figure 4, Figure 5 and Figure 6 indicate that the predicted results of the machine learning models depend strongly on the feature of the data in the training set. If the data in the training set cannot provide enough information for the data in the testing set (e.g., the anomalous data), it is hard to give reasonable or accurate predictions.

**Figure 7:**
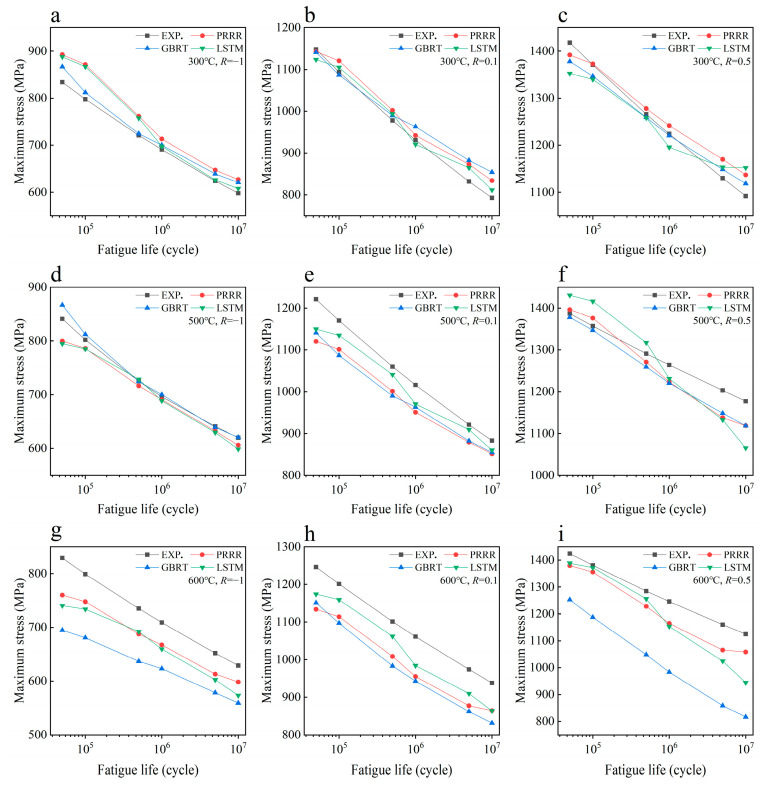
Predicted results of different machine learning models for the fatigue strength of the nickel-based superalloy under different conditions in case 3: (**a**) 300 °C, *R* = −1; (**b**) 300 °C, *R* = 0.1; (**c**) 300 °C, *R* = 0.5; (**d**) 500 °C, *R* = −1; (**e**) 500 °C, *R* = 0.1; (**f**) 500 °C, *R* = 0.5; (**g**) 600 °C, *R* = −1; (**h**) 600 °C, *R* = 0.1; (**i**) 600 °C, *R* = 0.5.

**Figure 8:**
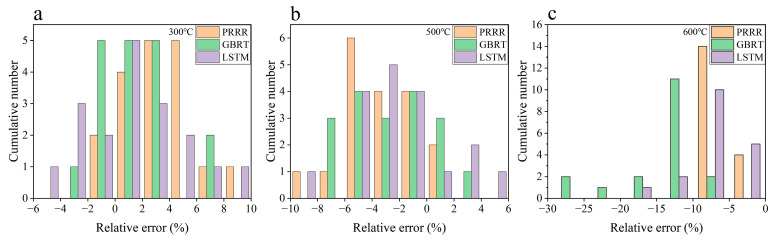
Relative errors of the predicted results to the experimental data for the different machine learning models in Figure 7. (**a**) 300 °C; (**b**) 500 °C; (**c**) 600 °C. The range of relative errors is the same between the same numbers on the horizontal axis.

Figure 7 shows the predicted results of the three machine learning models for the fatigue strength of the nickel-based superalloy at different temperatures and stress ratios in case 3. Figure 8 shows the relative errors of the predicted values to the experimental data for the different machine learning models. It is seen from Figure 7 and Figure 8 that all three machine learning models have strong predictive ability for the fatigue strengths at 300 °C and 500 °C under different stress ratios. The relative errors of the predicted values to the experimental ones are within 10%, as shown in Figure 8a,b, which indicates a satisfactory expectation from the machine learning models. However, the prediction accuracy of the fatigue strength is poor at 600 °C for the GBRT model, as shown in Figure 8c. Compared with the GBRT model, the predictive ability is acceptable for the PRRR and LSTM model, as shown in Figure 8c. The relative errors of the predicted results are all less than 10% for the PRRR, and most of the relative errors are less than 10% for the LSTM model. The difference of the prediction accuracy between the fatigue strengths at 300 °C and 500 °C and the ones at 600 °C is attributed to the influence of the anomalous data at 700 °C in the training set. The fatigue strength at the temperatures ranging from 23 °C to 600 °C shows a certain rule under different stress ratios, which is either close or presents a trend in general with the variation of temperature. While the trend of the fatigue strength at 700 °C is different from those at 23–600 °C. The temperature 600 °C in the testing set is near the temperature 700 °C in the training set. The anomalous data at 700 °C causes the side effects on the accuracy of the models generated by training especially for the GBRT model used in this paper, which results in the poor predictions for the fatigue strengths at 600 °C.

**Figure 9:**
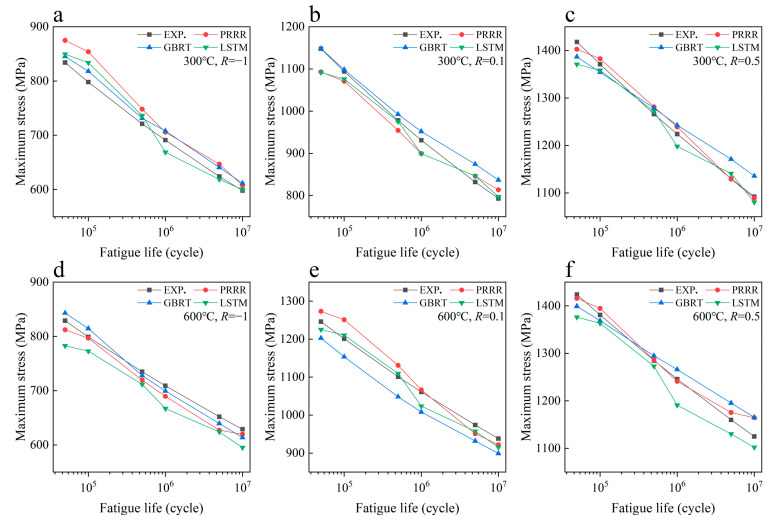
Predicted results of different machine learning models for the fatigue strength of the nickel-based superalloy under different conditions in case 4: (**a**) 300 °C, *R* = −1; (**b**) 300 °C, *R* = 0.1; (**c**) 300 °C, *R* = 0.5; (**d**) 600 °C, *R* = −1; (**e**) 600 °C, *R* = 0.1; (**f**) 600 °C, *R* = 0.5.

**Figure 10:**
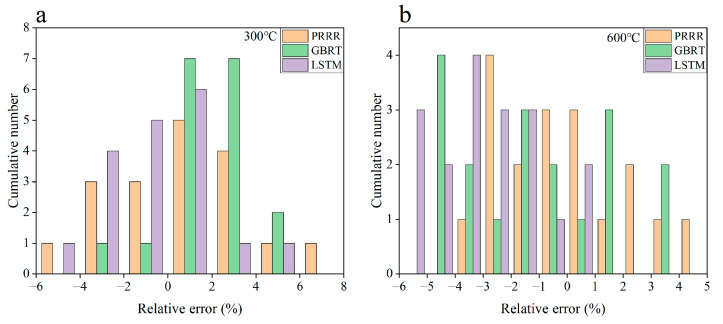
Relative errors of the predicted results to the experimental data for the different machine learning models in Figure 9. (**a**) 300 °C; (**b**) 600 °C. The range of relative errors is the same between the same numbers on the horizontal axis.

Figure 9 shows the predicted results of the three machine learning models for the fatigue strength of the nickel-based superalloy at different temperatures and stress ratios in case 4. Figure 10 shows the relative errors of the predicted values to the experimental data for the different machine learning models. A comparison of Figure 7 and Figure 8 with Figure 9 and Figure 10 indicates that the prediction accuracy of the fatigue strength at 600 °C is significantly improved for all three machine learning models. The relative errors of the predicted values are all within 6%, as shown in Figure 10b. The results in Figure 7, Figure 8, Figure 9 and Figure 10 indicate that if the anomalous data are included in the training set and the data in the training set are not enough, it is often hard to give accurate predictions by the machine learning method. By adding more normal data into the training set, the influence of the anomalous data on the prediction accuracy is weakened, and the prediction accuracy could be improved.

Table 2 shows the determination coefficient *R*^2^ between the predicted values and the experimental ones under the special temperatures by the three machine learning models with different training sets. The value of the coefficient of determination is calculated according to Equation (8), which could reflect the prediction accuracy of the model [45]. The closer the value of *R*^2^ to 1, the closer the predicted value to the experimental one. It is seen from Table 2 that, for the fatigue strengths at 400 °C in case 1, 300 °C and 500 °C in case 3 and 300 °C in case 4, the predictive ability is almost the same for the three machine learning models. While for the fatigue strengths at 700 °C in cases 1 and 2, as well as the fatigue strengths at 600 °C in cases 3 and 4, the PRRR is better than the GBRT and LSTM models. This indicates that the PRRR is the best one among the three machine learning models. The PRRR shows a good potential in dealing with the side effects of the data in the training set.

(8) $$R^{2}=1-\frac{\sum_{i=1}^{n}{\left(y_{i}-\hat{y_{i}}\right)}^{2}}{\sum_{i=1}^{n}{\left(y_{i}-\bar{y}\right)}^{2}}$$

where $y_{i}$ is the experimental value; $\hat{y_{i}}$ is the predicted value; and $\bar{y}$ is the average experimental value.

The above results indicate that the composition of data in the training set and the anomalous data have great influence on the predictive ability of the machine learning models. It is understandable that the machine learning method cannot give reasonable predictions for the anomalous data relative to the data in the training set. This is due to the fact that the essence of the machine learning method is data-driven, which is completely different from the physical models. In other words, the machine learning method is, to a large extent, a black box of which the physical meaning cannot be clearly explained. This is an inevitable fault in the application of the machine learning method. It is precisely because of the poor interpretability of the model that the machine learning method is limited in many specific fields, such as the fields of finance and healthcare, and so on. However, it is undeniable that, under some conditions, the machine learning method could play an important role in the prediction of fatigue performance and the anti-fatigue design of materials.

## 5. Conclusions

In this paper, the GBRT model, LSTM model and PRRR in machine learning are used to predict the fatigue strength of the nickel-based superalloy GH4169 under different temperatures, stress ratios and fatigue life. By dividing training sets and testing sets in different ways, the influence of the composition of data in the training set on the predictive ability is investigated for the machine learning method. This paper indicates that the machine learning method can give good predictions for the influence of various factors on fatigue strength by learning and training limited data, but the prediction accuracy strongly depends on the composition and characteristics of the training set. If the anomalous data are absent in the training set, it is hard to predict well the anomalous data even for the large amount of training data. When the anomalous data are in the training set, it will have a side effect on the predictive ability of the model generated by training. The influence of the anomalous data on the prediction of the normal data can be weakened with the increase in normal data in the training set. This paper also indicates that the PRRR shows the best prediction of the fatigue strength of the nickel-based superalloy among the GBRT model, LSTM model and PRRR. It is noted that this work is based on the fatigue strength data of the nickel-based superalloy GH4169 under different temperatures, stress ratios and fatigue life, which lacks a consideration of the relationship between the mechanism of influencing factors and fatigue strength. In future work, the role of physical parameters on fatigue performance under different loading conditions in machine learning models should be paid attention. More data and research are also needed to verify the reliability and practicability of machine learning methods due to the limited data of the present study.

## Figures and Tables

**Table 1:** Four different cases of training sets and testing sets of fatigue strength data.

	Case 1	Case 2	Case 3	Case 4
Training sets	23 °C, 300 °C, 500 °C, 600 °C	23 °C, 300 °C, 400 °C, 500 °C, 600 °C	23 °C, 400 °C, 700 °C	23 °C, 400 °C, 500 °C, 700 °C
Testing sets	400 °C, 700 °C	700 °C	300 °C, 500 °C, 600 °C	300 °C, 600 °C

**Table 2:** Determination coefficient *R*^2^ between the predicted values and the experimental ones under special temperatures for the four cases of different training sets and testing sets in Table 1.

Case	Temperature	Model	*R* ^2^	Temperature	Model	*R* ^2^
Case 1	400 °C	GBRT	0.980	700 °C	GBRT	0.459
		PRRR	0.994		PRRR	0.730
		LSTM	0.991		LSTM	0.330
Case 2	700 °C	GBRT	0.487			
		PRRR	0.730			
		LSTM	0.286			
Case 3	300 °C	GBRT	0.988	500 °C	GBRT	0.970
		PRRR	0.980		PRRR	0.965
		LSTM	0.980		LSTM	0.967
	600 °C	GBRT	0.536			
		PRRR	0.914			
		LSTM	0.902			
Case 4	300 °C	GBRT	0.990	600 °C	GBRT	0.984
		PRRR	0.988		PRRR	0.992
		LSTM	0.991		LSTM	0.984

## Data Availability

Not applicable.
